# The impact of early stages of COVID-19 on the mental health of autistic adults in the United Kingdom: A longitudinal mixed-methods study

**DOI:** 10.1177/13623613211065543

**Published:** 2022-01-27

**Authors:** Rebecca Bundy, Will Mandy, Laura Crane, Hannah Belcher, Laura Bourne, Janina Brede, Laura Hull, Jana Brinkert, Julia Cook

**Affiliations:** 1 University College London, UK; 2 King’s College London, UK

**Keywords:** adults, anxiety, autism spectrum disorders, COVID-19, depression, health services, mental health, qualitative research

## Abstract

We used mixed methods to learn about the nature and drivers of mental health changes among autistic adults in the United Kingdom during the early stages of the COVID-19 pandemic. In quantitative analyses, we examined the nature and predictors of change in depression, anxiety and stress, prospectively measured in 70 autistic adults at Wave 1 (just before the United Kingdom’s first lockdown) and Wave 2 (10–15 weeks into the United Kingdom’s first lockdown). Retrospective Wave 2 reports of mental health change were also analysed for these 70 participants. For the qualitative analysis, 133 participants (including the 70 from the quantitative analyses) provided reports on their experiences of the pandemic at Wave 2. In quantitative analyses, retrospective reports indicated that participants’ mental health worsened, but prospective data showed a different picture, with overall anxiety and stress scores reducing between Waves 1 and 2. Nevertheless, the mental health impact of the pandemic on autistic adults was variable, with a sizable minority reporting a significant decline in mental health. Qualitative analysis yielded four themes that contributed to mental health changes: (a) adjusting to changes to the social world, (b) living with uncertainty, (c) disruptions to self-regulation, and (d) barriers to fulfilling basic needs.

**Lay abstract:**

During the COVID-19 pandemic, high levels of depression, anxiety and stress have been reported in the general population. However, much less has been reported about the impact of COVID-19 on the mental health of autistic people. What we did: In the present study, we investigated how the mental health of autistic adults in the United Kingdom changed during the early stages of the COVID-19 pandemic. In total, 133 participants completed an online survey at two different time points. Of the 133 participants, 70 completed the survey at the first time point just before the onset of the national lockdown. This allowed us to look at changes in their mental health, from before the lockdown to 10 to 15 weeks during lockdown. All participants (133) told us about their experiences of the pandemic. What we found: While many autistic adults told us that their mental health worsened, people’s experience varied. For some autistic adults, aspects of mental health (e.g. anxiety, stress) actually improved. Participants also described social changes that had occurred, at home and in the outside world. They described feelings of uncertainty during the pandemic, and discussed how the pandemic had affected some of their previous coping strategies. Participants also told us about their difficulties in accessing healthcare services and food during the early stages of the pandemic. In our article, we discuss these findings and focus on what needs to change to ensure that autistic people are better supported as the pandemic continues.

A growing body of research has examined the impact of COVID-19 on mental health (e.g. Fancourt et al., 2021; Salari et al., 2020; Vindegaard & Benros, 2020; Xiong et al., 2020). This literature shows that subgroups of individuals have experienced higher rates of stress, anxiety and depression during the pandemic. Risk factors associated with mental health decline included being female, being a young adult, having pre-existing physical or psychiatric conditions, being unemployed or a student, living alone or having a child, having lower household incomes, living in urban areas, and frequent exposure to social media or news relating to COVID-19 (Fancourt et al., 2021; Salari et al., 2020; Vindegaard & Benros, 2020; Xiong et al., 2020).

Autistic people may have experienced especially severe challenges to their mental health and well-being during the pandemic (e.g. Oomen et al., 2021; Pellicano & Stears, 2020). First, as a group, autistic people are commonly exposed to several risk factors associated with pandemic-related mental health decline, such as high rates of pre-pandemic mental health problems (Lai et al., 2019, see also Bal et al., 2021), high rates of unemployment and poverty (Keen et al., 2016; Taylor et al., 2015) and increased risk of physical disabilities (Kinnear et al., 2020) and chronic diseases (Bishop-Fitzpatrick & Rubenstein, 2019; Cashin et al., 2018; Kinnear et al., 2020).

Second, some autistic characteristics may make dealing with the pandemic especially difficult. Many autistic people have a strong preference for sameness and predictability (Boulter et al., 2014), potentially making it challenging to tolerate the uncertainty that characterises life in the pandemic, including ever changing, externally imposed regulations. Indeed, a large European study, conducted in the early stages of the pandemic, found that autistic adults tended to report that COVID-19 had resulted in them becoming more stressed due to enforced changes to their daily routines (Oomen et al., 2021).

Third, disruption in access to services might have had a disproportionate effect on autistic people. Pre-pandemic, autistic people were more likely to need a range of healthcare services (Foley et al., 2018; Vohra et al., 2016) and have trouble accessing these (Mason et al., 2021; Nicolaidis et al., 2015; Vogan et al., 2017), compared with non-autistic people. Given the pressures placed on healthcare services during the pandemic, as well as the implementation of social distancing measures, the barriers to autistic peoples’ service access may have been further exacerbated. This notion is supported by reports from autistic adults of widespread disruption to their service access during the pandemic (e.g. Pellicano et al., 2021), with some evidence that this affected their well-being (Bal et al., 2021; Oomen et al., 2021).

Above, we have suggested that the COVID-19 may have had an overall negative effect on the mental health of autistic people. Nevertheless, the picture is likely to be complex and characterised by individual differences. While some studies have found evidence for an overall decline of autistic adults’ mental health in the pandemic (Oomen et al., 2021; Pellicano et al., 2021), other well-conducted longitudinal studies instead showed a picture of stability (Adams et al., 2021; Bal et al., 2021). Certain factors may protect autistic people against the negative effects of the pandemic. Given that autistic people report less social participation (Shattuck et al., 2011) and fewer close friendships (Baron-Cohen & Wheelwright, 2003; Liptak et al., 2011) compared with non-autistic people, it is possible that they may be less affected by the social distancing measures implemented to constrain COVID-19 transmission. Mental health might even have improved given reductions in social obligations. In line with this, compared with a non-autistic comparison group, autistic participants were more likely to report that the pandemic had helped reduce stress associated with social life and sensory overload (Oomen et al., 2021).

Another potentially protective factor for some autistic people during the pandemic has been the move to remote interaction. Remote working may have made employment more accessible and comfortable for some autistic people. Likewise, the move to telehealth in the delivery of services (Zhou et al., 2020) could have benefitted autistic individuals. Pre-pandemic, there was increasing interest in telehealth for autistic people as a plausible means to increase accessibility of healthcare provisions (Alfuraydan et al., 2020; Dahiya et al., 2020; Unholz-Bowden et al., 2020; Valentine et al., 2021). Although initial results showed some promise, issues such as adapting telehealth services to support autistic individuals with more complex needs (Tomlinson et al., 2018) meant that most reviews have concluded that further research is required. In addition, there is no strong evidence that telehealth improves service access for autistic people. As such, it will be valuable to investigate the effect of the pandemic-related move to telehealth for autistic people.

To summarise, there is reason to believe that the COVID-19 pandemic has had a significant negative impact on the well-being and mental health of autistic people. Nevertheless, it is likely that the pandemic’s impact on autistic people has been complex, variable and perhaps not unequivocally negative. Gaining an accurate picture of the impact of the pandemic on autistic people could inform service planning, identify where support should best be targeted, and highlight potential preventive measures that can protect the mental health of autistic people (e.g. in future pandemics). The present research seeks to understand better the effect of the pandemic on autistic adults’ mental health, using both quantitative and qualitative data from a two-wave, longitudinal survey. Specifically, we asked two questions. First, what was the impact of the early stages of the COVID-19 pandemic on the mental health of autistic adults in the United Kingdom? Second, what were the mechanisms whereby the pandemic affected UK autistic adults’ mental health?

## Method

### Design and participants

To take part, participants needed to be 18 years or older and either formally diagnosed or self-identified as autistic.^1,2^

### Context

On 23 March 2020, the United Kingdom entered a national lockdown. Many businesses and schools physically closed and moved online, and people were only permitted to leave the house for limited purposes, including food shopping, one form of daily exercise, urgent medical needs, and work (where necessary). To understand better the specific context of our participants in the United Kingdom, we gathered data on COVID-related restrictions in our Wave 2 survey. Most participants in the current study reported that events were suspended (*n* = 126 of 133, 94.7%), schools were physically closed to most children (*n* = 119, 89.5%) and non-essential shops were closed (*n* = 117, 88%). Restrictions were applied to non-essential movement and travel (*n* = 99, 74.4%), as well as activities outside (*n* = 71, 53.4%). Land borders were closed (*n* = 46, 34.6%), and people arriving in the country were required to self-quarantine (*N* = 73, 54.9%).

### Participant flow

All participants were initially recruited for a longitudinal online study (unrelated to COVID-19) on ‘autistic adults’ social behaviours, relationships and well-being’, via social media, autism support groups and the Cambridge Autism Research Database (CARD). These data were collected between February and April 2020 for 258 UK-based participants (Wave 1). Participants were then re-invited to complete a COVID-19-related survey (Wave 2) between May and July 2020. In total, 133 participants completed both waves, forming the current sample.

#### Quantitative analysis subsample

Wave 1 data collection spanned a period immediately before and after the start of the UK lockdown. Of the 133 participants who completed both waves, 70 provided Wave 1 data before the implementation of the lockdown in the United Kingdom. These 70 participants form our quantitative analysis subsample: their data were used for quantitative prospective and retrospective analyses. This is because we wanted to look at change using a pre-lockdown baseline and because we wanted prospective and retrospective analyses to be on the same sample to facilitate interpretation of any differences in findings.

#### Qualitative analysis total sample

The qualitative analysis in this mixed-methods study used the Wave 2 data from all 133 participants (see Table 1 for demographic information).

**Table 1.** Participant characteristics.

Characteristics *n* (%)		Total participants *N* = 133	Quantitative subsample *N* = 70	Qualitative only subsample *N* = 63	*t* test/odds ratio
Birth sex	Female	92 (62.2%)	58 (82.9%)	36 (57.1%)	2.14
	Male	39 (29.3%)	10 (14.3%)	27 (42.9%)	(1.05–4.44)
	Other (intersex and no sex)	2 (1.5%)	2 (2.9%)	0	
Gender identity^a^	Female	84 (63.2%)	53 (75.7%)	31 (49.2%)	1.82
	Male	38 (28.6%)	11 (15.7%)	27 (42.9%)	(0.95–3.48)
	Non-binary/Bigender	8 (6%)	5 (7.1%)	3 (4.8%)	
	Cisgender	6 (4.5%)	5 (7.1%)	1 (1.6%)	
	Other	5 (3.8%)	3 (4.3%)	2 (3.2%)	
	Gender neutral	4 (3%)	2 (2.9%)	2 (3.2%)	
	Transgender	1 (0.8%)	0	1 (1.6%)	
Age (years)	Mean	42.93	39.8	46.48	3.13***
	*SD*	12.76	10.2	12.76	
	Range	20.72	21–65	20–72	
Description of diagnosis	Autism	91 (68.4%)			
	Atypical autism	28 (21.1%)			
	Autism spectrum disorder	14 (10.5%)			
Diagnostic status	Formally diagnosed	124 (93.2%)	62 (88.6%)	54 (85.7%)	0.56
	Self-diagnosed	9 (6.8%)	8 (11.4%)	1 (1.6%)	(0.21–1.5)
Autism Spectrum Quotient–10 (AQ-10)	Mean	8.16	8.56	9.10	0.11
	Median	9	9	9	
	Mode	9	7	11	
	*SD*	1.61	1.88	1.92	
Living arrangements	At home with partner and/or children	64 (48.1%)	38	26 (41.3%)	0.64
	At home alone	35 (26.3%)	13	22 (34.9%)	(0.31–1.3)
	At home with parents and/or grandparents and/or siblings	19 (14.3%)	10	9 (14.3%)	
	At home with flatmates/friends	8 (6%)	5	3 (4.8%)	
	Other	6 (4.5%)	4	2 (3.2%)	
	In supported accommodation	1 (0.8%)	0	1 (1.6%)	
Highest level of education/qualification	Postgraduate university degree	47 (35.3%)	23 (32.9%)	24 (38.1%)	0.91
	Undergraduate university degree	34 (25.6%)	19 (27.4%)	15 (23.8%)	(0.50–1.64)
	Secondary/high school or equivalent	25 (18.8%)	14 (20%)	11 (17.5%)	
	Technical school/trade school/apprenticeship	15 (11.3%)	78 (11.4%)	7 (11.11%)	
	Other qualifications	12 (9%)	6 (8.6)	6 (9.5%)	
Current education & employment^b^	Full-time paid work	46 (34.6%)	23 (32.9%)	21 (33.3%)	0.80
	No employment, not looking for work	34 (25.6%)	18 (25.7%)	16 (25.4%)	(0.44–1.43)
	Part-time paid work	26 (19.5%)	11 (15.7%)	14 (22.2%)	
	Other	19 (14.3%)	9 (12.9%)	9 (14.3%)	
	Part-time education	15 (11.3%)	10 (14.3%)	5 (7.7%)	
	Working voluntarily	9 (6.8%)	6 (8.7%)	3 (4.8%)	
	Full-time education	8 (6%)	3 (4.3%)	5 (7.9%)	
	No employment, looking for work	6 (4.5%)	3 (4.3%)	3 (4.8%)	

Gender: M: Male, F: Female, N: Neutral, C: Cisgender, NB: Non-binary, AQ: Autism Quotient.

Statistical difference between participants in quantitative subsample (Wave 1) and qualitative (Waves 1 and 2) subsample only *t* test presented as *t*-statistics for continuous data and odd-ratios for categorical data.

a Category not mutually exclusive.

b Employment status prior to the pandemic.

*** *p* < 0.001.

Ethical approval for the study was obtained from the University College London Research Ethics Committee with an amendment submitted to collect data for Wave 2 during the pandemic.

### Materials

During the Wave 1 data collection, participants completed the online questionnaire that comprised demographic questions and the Autism Spectrum Quotient–10 (AQ-10), to better characterise our sample.

Participants also completed the Depression and Anxiety Stress Scales (DASS-21, Lovibond & Lovibond, 1995) during Waves 1 and 2 (to assess changes in mental health scores over time). The DASS-21 is thought to reliably assess depression (α = 0.93), anxiety (α = 0.84) and stress (α = 0.88; Park et al., 2020) in autistic people.

In addition to recompleting the DASS-21 at Wave 2, participants provided demographic information and answered bespoke questions about mental health effects of the pandemic (provided in the Supplemental Material; Table S1). In total, 19 COVID-related questions were included in this survey, divided into four sections: (a) access to service support, (b) mental health change, (c) hardships that have affected mental health, and (d) factors supporting mental health.

Of the 19 COVID-related questions, eight were open-ended, yielding the data used in our qualitative analysis. These open-ended questions asked participants to describe the following: how easy or difficult it had been to understand and access government information relating to COVID-19, the degree to which their needs had been met by services, how COVID-19 had impacted their mental health, how hardships had impacted them, factors that had helped their mental health during the pandemic, and whether their life had improved in any way because of the pandemic. The remaining 11 questions were either multiple choice questions or questions using rating scales, and covered lockdown measures, perceived mental health changes, and access to support services.

### Community involvement

Our team included autistic and non-autistic researchers.

### Data analysis

#### Quantitative data

To investigate the effects of the pandemic on mental health (anxiety, depression, stress), we used two quantitative approaches with data from the 70 participants who provided Wave 1 data before the start of the UK lockdown. First, we analysed their retrospective reports, collected at Wave 2, of perceived mental health changes during the pandemic. Second, we conducted longitudinal analyses of prospective DASS-21 subscale data, collected at Waves 1 and 2. In these prospective analyses, we examined change at the group level, testing for changes in mean scores on the DASS-21 over time using within-person *t* tests. We also examined change at the individual level on each subscale of the DASS-21, using the reliable change index (RCI) and investigating clinically significant change (CSC). This allowed us to identify individuals whose DASS-21 scores (a) got worse, (b) did not change, or (c) improved. The RCI provides a standardised score reflecting statistically reliable changes and direction of change over time for each participant (Jacobson & Truax, 1991). An RCI ⩾ 1.96 indicated an increase in mental health symptoms over time, whereas ⩽−1.96 indicated a reduction. Participants with an RCI between −1.96 and 1.96 did not show a reliable change in mental health symptoms over time. CSC indicates whether an individual moved across the clinical threshold on a relevant subscale of the DASS-21, moving either in or out of the clinical range or showing no change. For the purpose of the study, for each DASS-21 subscale, scores in the moderate to extreme range were considered to be above the clinical threshold.

We also examined the drivers of mental health change using our longitudinal data. For each participant, change scores were calculated for each DASS-21 subscale by subtracting Wave 1 from Wave 2 scores. Thus, a positive DASS-21 change score indicated an increase in mental health difficulties. Then, partial correlations examined associations between (a) DASS-21 change scores for depression, anxiety and stress; and (b) demographic factors and data on COVID-related impacts as reported at Wave 2 (e.g. access to support, financial impact, maintaining routines and leisure activities), controlling for the relevant baseline DASS-21 subscale (collected during Wave 1). Where we identified significant relationships, predictors were simultaneously entered into linear regression models with DASS-21 change scores as the outcome variable to identify which predictors uniquely contributed to change in DASS-21 score. In these models, we always controlled for the relevant baseline (Wave 1) DASS-21 score and assumptions of independence of errors were met.

#### Qualitative data

Qualitative data were analysed using reflexive thematic analysis (Braun & Clarke, 2006, 2013, 2019), which allowed for patterns of meaning to be drawn across the data. Analysis was conducted from a critical-realist framework, meaning participants’ accounts were taken as being true to them, as well as impacted by factors from wider social contexts (Braun & Clarke, 2013; Willig, 2013). An inductive approach was used, whereby themes were identified at a semantic level (i.e. themes were strongly linked to the data, rather than being driven by preconceived analytic assumptions of the researcher; Braun & Clarke, 2020). Analysis was led by R.B., who received regular input at all stages from J.C. and the wider research team. The analysis involved recursively moving through the data by reading and re-reading responses, while simultaneously making notes of emerging patterns. Extracts of data were then assigned codes, which were revisited by RB and JC separately and revised by RB. Codes were then organised into broader analytical themes. Finally, a negative case analysis was undertaken to highlight any data that contradicted the identified themes, to strengthen the rigour of the analyses (Tenzek, 2017).

Regarding positionality, all authors view autism within a social model, reflecting the principles of developmental psychopathology. Crucially, this approach places emphasis on the need to modify the environments in which autistic people exist, rather than placing sole emphasis on the need for autistic people to adapt to fit current structures in society (e.g. Mandy et al., 2016; Mandy & Lai, 2016). This positionality is likely to affect which questions were asked and how data were interpreted.

## Results

### Quantitative results – changes in mental health during the pandemic

#### Retrospective reports from Wave 2

**Figure 1.**
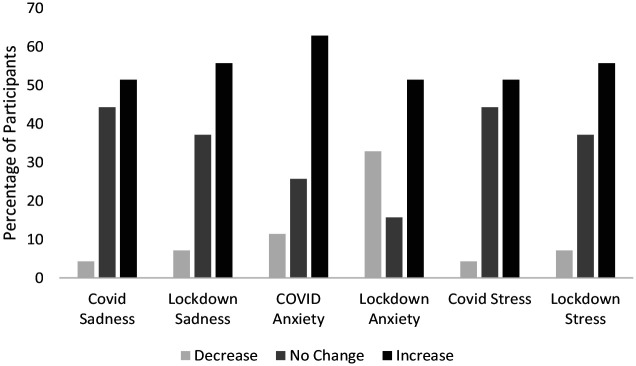
Participants’ retrospective perceptions of changes in anxiety, stress and sadness as a result of the COVID-19 virus and lockdown measures (*N* = 70). Retrospective experience assessed on the COVID-19 and lockdown questions on changes in anxiety, depression and stress.

At Wave 2, participants retrospectively reported how they felt the pandemic had changed their levels of anxiety, stress and sadness (see Figure 1). More than half of the participants reported increases in sadness, anxiety and stress related to COVID-19 and lockdown restrictions.

#### Prospective reports from Waves 1 and 2

Compared with the retrospective data, a somewhat different picture emerged when analysing the longitudinal DASS-21 data (see Table 2). At a group level, there was a tendency for DASS-21 scores to reduce (anxiety, stress) or stay the same (depression) between Waves 1 and 2. Our individual-level analyses, however, suggest more diversity in mental health changes during the pandemic. For example, even though overall anxiety and stress scores declined, a significant minority of participants (anxiety = 20%, stress = 21%) experienced a reliable increase in these difficulties. Regarding depression, the overall lack of change in the group mean over time belies the fact that around a third improved significantly (36%) while another third (36%) showed an increase in depression between Waves 1 and 2. As shown in Table 2, our index of CSC confirmed variability across participants. A majority of the participants scored in the clinical range at one or both time points. Around half of the participants showed clinically significant stress scores before and during the pandemic (48.6%), whereas slightly fewer participants showed clinically significant depression and anxiety scores at both time points (34.7% and 38.6%, respectively).

**Table 2.** Descriptive statistics and differences at DASS-21 at Waves 1 and 2 and changes over time with reliable change index.

DASS-21 (*N* = 70)	Wave 1 *M* (*SD*)	Wave 2 *M* (*SD*)	*t* test	*p* value	Effect size	95% CI	Change (Wave 2 – Wave 1) *M* (*SD*)	CSC *n* (%)	RCI *n* (%)
Depression	19.63 (13.10)	19.42 (12.93)^a^	0.22	0.83	0.03	[−0.21, −0.26]	−0.49 (18.46)	Clinical classification: 24 (34.7%) Clinical classification change to non-clinical: 16 (23.2%) Non-clinical change to clinical classification: 17 (24.6%) Non-clinical range: 12 (17.4%)	Decrease: 25 (35.7%) No change: 19 (27.1%) Increase: 25 (35.7%)
Anxiety	16.34 (9.64)	12.26 (10.86)	2.36	0.02**	0.28	[0.04, 0.52]	−4.09 (14.47)	Clinical classification: 27 (38.6%) Clinical classification change to non-clinical: 25 (35.7%) Non-clinical change to clinical classification: 9 (12.9%) Non-clinical range: 9 (12.9%)	Decrease: 27 (38.6%) No change: 29 (41.4%) Increase: 14 (20%)
Stress	26.23 (8.59)	22.74 (10.02)	2.24	0.03**	0.27	[0.03, 0.51]	−3.49 (13.00)	Clinical classification: 34 (48.6%) Clinical classification change to non-clinical: 19 (27.1%) Non-clinical change to clinical classification: 10 (14.3%) Non-clinical range: 7 (10%)	Decrease: 25 (35.7%) No change: 30 (42.9%) Increase: 15 (21.4%)

DASS-21: Depression and Anxiety Stress Scales (Lovibond & Lovibond, 1995). CI: confidence interval; RCI: reliable change index; CSC: clinically significant change.

Means and standard deviations presented for Waves 1 and 2, change *p*, Cohen’s *d* and 95% CI. RCI and CSC presented with frequencies and percentages.

a Data missing for the depression subscale for one participant during Wave 2.

** *p* < 0.01.

Given the somewhat contrasting findings between our prospective and retrospective data, we looked at how these different indices of mental health change were associated. Partial correlations to control for baseline mental health difficulties on the DASS-21 indicated that retrospective and prospective data were moderately correlated (*r*s between 0.20 and 0.53, see Supplemental Material, Table S3).

### Quantitative results – factors associated with changes in depression, anxiety and stress in longitudinal data

To assess factors associated with mental health changes, partial correlations were run for DASS-21 change scores, demographic factors, AQ-10 and COVID-19 variables while controlling for pre-pandemic (Wave 1) DASS subscale scores (see Table 3). Then, the relevant DASS-21 pre-pandemic scores and significant variables in the correlation analyses were entered into three multiple linear regression models to ascertain their independent effects on changes in anxiety, depression and stress, respectively (see Table 4). Frequencies of COVID-19 variables included in the models can be found in the Supplemental Material, Table S2.

**Table 3.** Partial correlations for DASS-21 change scores, demographic factors, AQ-10 and COVID-19 variables.

	Change in DASS-depression from Waves 1 and 2 (controlling for pre-pandemic DASS-depression scores)	Change in DASS-anxiety from Waves 1 and 2 (controlling for pre-pandemic DASS-anxiety scores)	Change in DASS-stress from Waves 1 and 2 (controlling for pre-pandemic DASS-stress scores)
Sex	−0.10	0.08	0.15
Age	0.001	0.16	0.09
AQ	−0.02	0.06	0.13
Co-occurring conditions	0.07	−0.01	0.05
Living arrangements alone vs living with others	0.22	0.17	0.07
Change in access to support	0.40**	0.27	0.34*
Access to basic needs	−0.20	−0.11	−0.05
Financial impact	0.24	0.08	0.11
Regular exercise	−0.32*	−0.23	−0.13
Leisure activities alone	−0.34*	−0.13	−0.05
Engaging in social activities	−0.49***	−0.45***	−0.31*
Self-care for mental health	−0.34*	−0.08	−0.01
Establishing a regular routine	−0.31*	0.05	0.08
Uncertainty about lockdown measures	0.14	0.33*	0.32*
Changes to routine	0.11	0.18	0.34*
Leisure activities with others	−0.27	−0.20	−0.12

DASS-21: Depression and Anxiety Stress Scales; AQ: Autism Quotient–10.

Partial correlations controlling for the relevant DASS-subscales for depression, anxiety and stress pre-pandemic (Wave 1) between changes in the DASS-21 subscales, calculated as a difference score for data collected before and during the pandemic, for depression, anxiety and stress and age, sex, co-occurring conditions (frequency of having co-occurring physical or mental health conditions), living arrangements (living alone or with others), AQ, changes in access to support, access to basic needs (such as medicine or food), financial impact of the pandemic, engaging in regular exercise, leisure activities alone, leisure activities with others (social activities), self-care for mental health (e.g. engaging in yoga or mediation), and having developed a new routine as a result of the pandemic, experiencing uncertainty, changes in routine are negatively impacting well-being.

* p < 0.05; **p < 0.01; ***p < 0.001.

**Table 4.** Summary of the results from the linear regression models.

	*B*	*SE*	β	*t*	*p*	Collinearity statistics	
						Tolerance	VIF
Model 1 Depression Changes: Adjusted *R*^2^ = 0.70, *F*(7, 42) = 16.94, *p* < 0.001							
(Intercept)	27.88	5.12		5.44	**<0.001**		
Wave 1 DASS-21 depression	−0.86	0.13	−0.59	−6.91	**<0.001**	0.85	1.18
Access to support	7.56	3.41	0.20	2.22	**0.03**	0.74	1.35
Regular exercise	−5.65	3.29	−0.15	−1.72	0.09	0.80	1.25
Leisure activities alone	−0.66	3.82	−0.02	−0.17	0.87	0.81	1.24
Social engagement	−10.15	3.21	−0.27	3.16	**0.003**	0.84	1.19
Self-care	−2.14	3.39	−0.06	−0.63	53	0.79	1.26
Established a new routine	−7.89	3.13	−0.21	−2.52	**0.02**	0.89	1.12
Model 2 Anxiety Changes: Adjusted *R*^2^ = 0.53, *F*(3, 65) = 26.68, *p* < 0.001							
(Intercept)	11.68	2.88		4.06	**<0.001**		
Wave 1 DASS-21 anxiety	−0.93	0.13	−0.62	7.28	**<0.001**	0.94	1.06
Social activities	−7.90	2.45	−0.28	−3.23	**0.002**	0.95	1.05
Uncertainty about lockdown measures	5.78	2.50	0.19	2.31	**0.02**	0.98	1.02
Model 3 Stress Change: Adjusted *R*^2^ = 0.47, *F*(5, 46) = 10.2, *p* < 0.001							
(Intercept)	13.11	5.65		2.32	**0.025**		
Wave 1 DASS-21 stress	−0.83	0.15	−0.57	−5.48	**<0.001**	0.95	1.05
Social activities	−6.11	2.69	−0.25	−2.28	**0.03**	0.89	1.13
Access to support	3.26	2.99	0.13	1.09	0.28	0.72	1.39
Uncertainty about lockdown measures	2.88	3.06	0.11	0.94	0.35	0.78	1.29
Disruption in routine	6.59	4.45	0.17	1.48	0.15	0.79	1.26

Statistics for each model presented on changes in depression, anxiety and stress as a result of the pandemic, coefficients (B), Standard error (*SE*), standardised beta values (β), t-statistics (*t*), significance level (*p* < 0.05, presented in bold) and the confidence interval (CI) for the coefficient and collinearity statistics with the tolerance and Variance Inflation Factor (VIF) for collinearity.

In the partial correlation analyses, an increase in DASS-21 depression scores was associated with reduced access to support services (while controlling for pre-pandemic depression scores). A decrease in depression scores was associated with greater engagement in social activities, self-care for mental health (e.g. yoga or meditation), maintaining a routine, doing leisure activities, and regular exercise. When these were simultaneously entered into a regression model, the pre-pandemic DASS-depression score, change of access to support services, having a routine and engaging in social activities were each uniquely predictive of changes in depression scores (see Table 4).

Increased anxiety between Waves 1 and 2 was associated with uncertainty about lockdown measures, while controlling for baseline (Wave 1) DASS-21 anxiety levels. Reduced anxiety was associated with engaging in social activities. As shown in Table 4, when these significant associations and baseline anxiety levels were entered simultaneously into a regression model, DASS-anxiety pre-pandemic scores, engaging in social activities and uncertainty each significantly predicted changes in anxiety symptoms.

Increased stress between Waves 1 and 2 was associated with higher pre-pandemic scores in the DASS-stress subscale, changes in access to support services in the correlation analyses, uncertainty about lockdown measures and changes in normal routine when controlling for stress at Wave 1. In the regression (see Table 4), DASS-21 stress pre-pandemic scores and taking part in social activities uniquely predicted changes in stress.

#### Qualitative analysis

**Figure 2.**
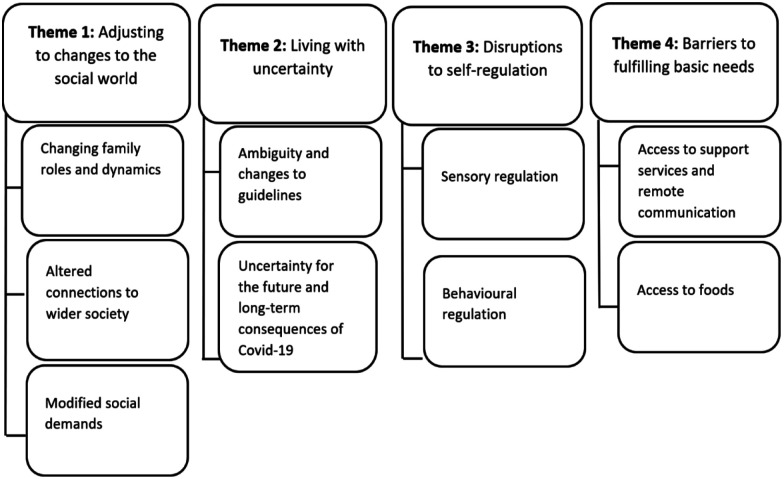
Themes and subthemes of how the COVID-19 pandemic affected the mental health of autistic adults.

Using thematic analysis, four overarching themes were identified: (a) adjusting to changes to the social world, (b) living with uncertainty, (c) disruptions to self-regulation and (d) barriers to fulfilling basic needs, each with several subthemes (see Figure 2). Illustrative data extracts for each theme (organised by subtheme) are provided in Tables 5 to 8. Participants are identified via numbers included after quotes.

**Table 5.** Illustrative data extract for Theme 1: Adjusting to changes to the social world.

Changing family roles and dynamics	‘Time spent with my family doing things together can be lovely – there are no other people I relate to so well and enjoy being with so much, although I still get exhausted through the contact. I love the feeling of being a network rather than an individual with them’ (52). ‘The children have been distressed, anxious and have found it too hard to cope with not knowing what is going to happen and we have had to cope with very distressed behaviours and increased instances of self-harm’ (45). ‘Increased time spent around family has caused me extra stress and anxiety as well as feelings of guilt, worthlessness and anger’ (123). ‘I am the carer, the one who puts people back together so I have to seem strong and not dissolve – but inside I do. I also have constant housework to do which is not valued – it’s unrelenting. I feel invisible. I feel exhausted’ (52). ‘The main concern was taking care of my parents. I am not a natural caregiver so it was a steep learning curve for me to adjust to having to cook and clean for them at first. My older sister isn’t autistic but she works as a senior nurse so wasn’t allowed to visit us and help out like she normally would have’ (114). ‘Having zero time at home alone. My wife is at home all day so [. . .] I have no down time to process the day. Just before lockdown the OT [Occupational Therapist] in the autism service encouraged me to have time alone each day, especially when I get in from work. Lockdown is making that impossible. Always anticipating an interruption to my thoughts or whatever (meaningful or meaningless) task I am doing’ (59).
Altered connections to wider society	‘This has caused loneliness and a sense of meaninglessness in my life as despite my social anxiety I strongly want to feel connected to people, something which the current isolation has denied me’ (123). ‘Practically all my coping strategies prior to COVID-19 involved other people, or travelling to places. All of that has been prevented by lockdown measures and I don’t know how to cope anymore. [. . .] Trouble is, I can’t get someone to hold my hand so I feel safe until I learn how things have changed and develop new ways to cope. I feel trapped, imprisoned alone’ (10). ‘I’ve had a couple of in-person visits from one of my partners since lockdown started – this contravenes lockdown rules but was necessary due to the severity of my mental health, which was causing me to self-harm due to despair at the lack of contact, and was pushing me close to suicide’ (10). ‘Often I struggle on my own and feel bad I can’t just “do normal” like other people. It’s validating to be struggling alongside others for a change’ (90). ‘It has taught me that the further I am from neurotypical people, the stronger my mental health is!’ (63).
Modified social demands	‘The pressure of having social engagements I’d rather avoid has gone! [. . .] I can stay at home without guilt – in fact I’ve been told to stay here!’ (52). ‘I have been much more aware of my masking when I have had to go into a work situation and realised just how much I do this and have been able to relax it during lockdown’ (39). ‘I have become anxious about leaving the house (something I have always found slightly difficult especially if I haven’t left the house at least every other day). Being in lockdown has meant being at home much more and this has made my anxiety about going out much worse. I have not felt able to go out on my own’ (118). ‘I don’t mind being in contact with other people but they tend to be on the phone for too long, and that can take its toll; I prefer short and often, they prefer long and not very often’ (6).

**Table 6.** Illustrative data extract for Theme 2: Living with uncertainty.

Ambiguity and changes to guidelines	‘There were written articles online, but every single one contradicted each other and the rules were never consistent. It made me extremely anxious, angry and worried that I weren’t following the correct procedures and rules, and that I might get in trouble by the police if I went outside for any reason. So I completely isolated myself in my house because I was too scared to get in trouble. This was very hard on my mental health’ (110). ‘It takes a lot out of my mental energy and adds to my cognitive load because I am always on edge and alert trying to figure out what the next thing to do is, or what the right thing to do’ (9). ‘Having to think extra things when I have had to go out (executive functioning) about protective measures like hand washing, not touching my face, taking hand sanitiser with me, keeping two metres apart from others can be tiring’ (39). ‘I am very distressed by people getting more lax about social distancing outdoors, even though I don’t feel I’m at high risk from COVID-19. I’m more upset that people aren’t doing what they should be which makes me anxious and upset’ (90).
Uncertainty for the future and long-term of COVID-19	‘I am fearful of how I will cope when I need to return to work due to the amount that will have changed. It will be difficult to manage and I don’t cope with change well’ (135). ‘I don’t know if people will see my degree as lesser due to finishing online or if I will be able to find any good employment afterwards [. . .] I wonder if it’s even worthwhile trying anymore’ (56).

**Table 7.** Illustrative data extract for Theme 3: Disruption to self-regulation.

Sensory regulation	‘I have not had any meltdowns since lockdown. I believe this is because I am working from home, no commute, no bus, no open plan office, no shopping centres. No sensory overload’ (126). ‘Noise from the neighbours being constantly home and in their garden, having parties, screaming kids etc. means having an open window increases the noise, but closing it means I am too hot. The noise and heat both overload my senses and I feel like screaming’ (31).
Behavioural regulation	‘My routine has changed dramatically and it has really thrown me. It has taken me many weeks to try and establish a new routine with when to go to bed, when to get up, how to work from home, how to work at school safely looking after the key worker children. And now it has all changed all over again. Every time it changes, it brings new anxiety’ (9). ‘I have enjoyed spending time in my garden and found a new enjoyment in nature’ (41). ‘My employer has put on be-well webinars for us to learn about homeworking, mental well-being, nutrition exercise etc., which inspired me to get into good habits with taking exercise, having a routine, switching off, going for walks, vitamins’ (126). ‘Some of my special interests have just stopped for the foreseeable future. This made it the most hard for me to adjust’ (12).

**Table 8.** Illustrative data extract for Theme 4: Barriers to fulfilling basic needs.

Access to support services and remote communication	‘Add the existing problems with waiting lists to the huge backlog that will result from the lockdown and nobody is going to get adequate care for a long time’ (13). ‘It feels like too much to deal with, made worse by no guidance or support services at all. We are trapped and frightened and feel entirely alone’ (45). ‘I also found video calls very tiring because there is so much noise and so much going on, trying to follow the conversations are really hard, especially with computer lag etc.’ (9). ‘Video calling and phone calls aren’t helpful for me due to the level of eye contact, appropriate conversation spacing and other skills required to maintain a decent level of social interaction. I find these just make me more stressed and anxious afterwards than when I began’ (104). ‘I have also been unable to continue with psychotherapy for depression and complex trauma as I couldn’t tolerate video therapy (I felt the human connection and sense of safety were lacking, both of which are incredibly important for me)’ (123).
Access to foods	‘I have an eating disorder, which is common in able autistic women, and being unable to get foods I rely on or brands I need is difficult, as is the limit of 3 of any one item’ (90).

### Theme 1: adjusting to changes to the social world

This theme refers to COVID-19-related social changes experienced inside and outside the household.

#### Sub-theme 1: changing family roles and dynamics

Participants described various ways that household dynamics had changed during lockdown. Family played a central role in the lives of many, and for some, the additional time spent together engaging in shared activities strengthened their bonds. Due to the importance of loved ones, many of the participants expressed concerns about loved ones becoming unwell with COVID-19 and how the pandemic had affected their lives. At the same time, increased time spent together resulted in family disputes and household disconnect more frequently occurring, which negatively affected participants’ mental health. Some participants also recognised challenges associated with changes to household dynamics, including increased responsibility. The ‘caring-role’ and need to portray external strength to protect others often affected participants’ own mental well-being. For others, the pandemic brought the need to adopt entirely new caring roles for vulnerable people in their household. In some participants, this was amplified by the absence of others who may ordinarily share such responsibilities. Adjusting to new roles was challenging and at times contributed to heightened anxiety. Challenges for many were often exacerbated in the absence of adequate ‘alone-time’, which was a vital form of coping through self-regulation.

#### Subtheme 2: altered connections to wider society

The pandemic altered most participants’ sense of connectedness, in various ways. For the majority, reduced in-person contact resulted in loss of connection to communities and social groups that participants had worked hard to build, and the desire to reconnect to these communities was associated with distress, loneliness and isolation. Prior to lockdown, many relied heavily on social support to manage their daily lives, the absence of which left participants fearful of how they would cope with the ongoing challenges of the pandemic. For a few participants, the need for social connection was felt so extremely that it resulted in self-harm and suicidal ideation, requiring them to break lockdown rules.

Other participants, however, felt the shared challenges of the pandemic contributed to heightened feelings of connection to others. These participants were comforted by others’ efforts to reconnect with them, which they felt had been encouraged by the pandemic. The experiences of shared struggles elicited feelings of validation for some participants, partly due to prior experiences of marginalisation. Entirely in contrast with this were a small number of participants that felt no desire to connect to wider society. These participants noticed a sharp improvement in their mental health considering altered connections.

#### Subtheme 3: modified social demands

While some participants felt a strong desire to connect with others during these times, many recognised that some of the social demands that had existed outside the home (e.g. workplace, school) prior to lockdown had negatively affected them and appreciated the relief. Fewer in-person social demands meant participants did not have to camouflage, mask, or ‘care about how to act, or what other people think’ (56) as much. However, some recognised that the forced social avoidance of lockdown exacerbated fears of socialising and raised concerns about having to reintegrate back into society when lockdown eased.

Alternatively, due to increased reliance on remote technology during lockdown, a subset of participants commented on feeling heightened pressures to socialise relative to pre-lockdown. Little time to recharge between interactions was associated with feelings of fatigue for these participants, who recognised the importance of managing the frequency and length of remote interactions.

### Theme 2: living with uncertainty

This theme encapsulates the level of uncertainty felt throughout the pandemic, focusing specifically on rules and guidelines, as well as long-term consequences of the pandemic.

#### Subtheme 1: ambiguity and changes to guidelines

Although some participants described feeling distressed by the perceived lack of time to prepare for lockdown, many found information presented at the start of lockdown clear and easy to understand. As lockdown began to ease, participants found rules and guidelines increasingly difficult to understand due to the lack of clarity of information across sources. Many described a duty to ‘obey to the letter’ (8), but the ambiguity of messages often left them unable to do so, which resulted in extreme distress, and fear of breaking rules. As a result, some participants further isolated themselves.

Changes to government rules and recommendations were also felt to be cognitively demanding due to participants having to remain vigilant to changes. Some described increased demands placed on their executive functions, for example, when making plans to leave the house. Not only was there concern about participants’ personal understanding of guidelines, but how well others understood them and followed them. This was often associated with emotional distress.

#### Subtheme 2: uncertainty for the future and long-term consequences of COVID-19

Uncertainty about job security, healthcare, education and more broadly ‘how life will differ’ (42) in the long-term, contributed to stress for many. Some questioned their ability to adapt back to ‘normality’ in the future due to the extent to which things had changed. For participants who were studying during lockdown, disruption to the way in which courses were taught alongside uncertainty about employment trajectory, left them fearful of the long-term consequences of the pandemic.

### Theme 3: disruptions to self-regulation

This theme encompasses changes that occurred to self-regulation, including sensory regulation and behavioural regulation.

#### Subtheme 1: sensory regulation

Due to closures and restrictions during lockdown, participants described their sensory worlds changing. Some noticed a reduction in sensory input and greater control over their sensory environment, which positively affected their ability to cope. At the same time, participants recognised challenges of managing their sensory environment at home. Due to more people staying at home, some experienced increased noise pollution in neighbourhoods and households during lockdown. This was further compounded by limited space to escape from the noise to self-regulate.

#### Subtheme 2: behavioural regulation

The ability to establish structured routines was helpful for many in managing the multifaceted stressors of the pandemic. When achieved, some felt more in control of routines due to fewer disruptions from external factors. At the same time, many participants described challenges maintaining routines in the face of frequent and often unexpected changes. For many, deviation from normality led to anxiety, and having to learn frequently to adjust to such changes negatively affected their mental health. For those that could access their routines, there was more time to engage in hobbies and special interests of both solitary nature and with households. Increased appreciation for nature was found by many participants over this period, alongside ‘self-care’ activities, including mindfulness, journaling or exercising.

For some participants, increased reliance on remote communication provided greater structure, consistency and control over chosen social outlets and offered opportunities, such as workshops, webinars and online exercise classes, that were ‘free from travel hassles’ (132) and supported their well-being. However, for other participants, engaging in special interests was challenging due to restrictions, which left them feeling a loss in their ‘purpose in life’ (88), leading to worsened mental health.

### Theme 4: barriers to fulfilling basic needs

This theme refers to participants’ ability to fulfil basic needs of accessing healthcare and food during the pandemic.

#### Subtheme 1: access to support services and remote communication

Experiences of accessing healthcare were variable. Long-standing feelings of services not meeting the needs of the autistic community were felt to have been exacerbated in the current climate. Many struggled with waiting times for healthcare services and/or felt concerned about continuity of their healthcare in the future. Participants with co-occurring health conditions experienced challenges accessing routine medications, which, compounded by inadequate support, contributed to a heighted sense of vulnerability.

There was general recognition that lockdown had increased reliance on remote interactions to access service support and sustain social connections during the pandemic, including, texts, phone calls, emails, and video calls. Several participants were offered remote support services. While a minority felt well supported by this, for the majority, remote support appeared to come with a new set of difficulties. Many described challenges associated with sensory aspects, such as background noise, which made conversations more challenging to process.

Video calls were associated with heightened pressure on nonverbal communication, including eye contact and conversational cues, and challenges interpreting body language. In addition, some participants noticed feeling self-conscious when using video calls, due to having to view themselves on screen, and reported feeling a lack of control over who was watching them on the other end.

Due to these challenges, some participants declined offers of remote therapy with concerns about it being inappropriate or inaccessible, and opted to wait for in-person contact to resume. Of participants that engaged, some found video calls a poor replacement to in-person contact, due to being ‘too unpleasant and cold’ (123) or feeling unsafe, which led to disengagement from service support entirely.

#### Subtheme 2: access to foods

Some participants struggled with changes to their food routine. This included shopping at different places and needing to purchase new brands or foods due to limitations. Owing to this, as well as general emotional upset, some described a reduction in their food intake, which resulted in an exacerbation of eating disordered behaviour for a few.

## Discussion

We investigated the mental health impact of the early stages of the COVID-19 pandemic on autistic adults across the United Kingdom. Combining quantitative and qualitative data yielded insights into (a) the nature of changes for autistic adults in depression, anxiety and stress in the early stages of the COVID-19 pandemic, and (b) the factors that influenced these mental health changes.

### What impact did the COVID-19 pandemic have on depression, anxiety and stress in autistic adults?

When using quantitative data to address this question, we received contrasting answers depending on whether we used retrospective reports or prospective, longitudinal data. When asking participants at Wave 2 to retrospectively report on their pandemic-related mental health changes over the past few months, most reported that their levels of sadness, anxiety and depression worsened. However, when we looked at changes in mental health scores taken at Wave 1 (before lockdown was imposed) and at Wave 2 (10–15 weeks into lockdown) for these same participants, overall levels of anxiety and stress appeared to have fallen, while average levels of depressive symptomatology stayed the same.

When comparing prospective and retrospective measurements of the same phenomenon, disagreement is common (e.g. Ralph et al., 2020). In such cases, it is logical to privilege the findings from prospective data, as retrospective reports are more subject to bias, particularly when individuals are asked to recollect complex and subjective phenomena such as emotional states over periods of several months. We must not discount subjective accounts, and it is important to acknowledge people’s perceptions that their mental health has worsened. However, from these data, we cannot conclusively claim evidence of an overall increase in mental health problems among UK autistic adults in the early stages of the pandemic. In fact, our prospective data suggested that overall levels of anxiety and stress may have fallen during this period. These findings are somewhat compatible with longitudinal investigations of US-based autistic people during the pandemic, which found no overall decline in mental health (Adams et al., 2021; Bal et al., 2021). Our findings also fit with a UK prospective study of more than 70,000 adults, which observed decreased mental health problems during the early stages of lockdown (Fancourt et al., 2021).

Nevertheless, compared with our group-level findings about changes in average levels of mental health symptoms, we consider our individual-level analysis of reliable and CSC to give a fairer picture of pandemic-related mental health changes. Our retrospective and prospective data demonstrated that reactions to the pandemic were diverse, with some autistic adults showing improvements, others showing no change and others demonstrating declines in mental health. This variability of mental health response to COVID-19 has been consistently observed in autistic (e.g. Adams et al., 2021) and non-autistic (e.g. Pierce et al., 2020) people. In the face of such variability, the crucial question becomes, what factors determine better or worse outcomes? It is this matter that we consider next.

### What factors influence pandemic-related mental health changes in autistic adults?

From both our qualitative and quantitative data, the pandemic appeared to have both beneficial and negative effects on autistic adults (e.g. sometimes a factor was beneficial to one person, but problematic for another). Next, we consider positive influences suggested from our data, before considering potential negative influences.

### Pandemic-related promoters of mental health

Our quantitative data showed that having social support and the ability to carry out leisure activities alone were associated with positive mental health changes in the early stages of the pandemic. Our qualitative analyses support this, with participants reporting that connections with family and friends were crucial to their well-being: challenging the unhelpful stereotype that all autistic people prefer to avoid social connections. Furthermore, our qualitative data confirmed that many autistic people valued the opportunity to pursue their interests in lockdown, and this promoted their well-being.

Sometimes lockdown helped reconfigure the environment in a way that was more accommodating of autistic people’s natural preferences. From our qualitative data, it was evident that relief from social pressures due to fewer in-person social demands led to an improvement in anxiety for many, and alleviated feelings of guilt that were previously associated with an avoidance of social occasions. Regarding this, some participants felt reductions in the need to socially camouflage or mask also contributed to general mental health improvements. Camouflaging has been associated with mental health challenges including anxiety, depression, and higher rates of suicidality (Cassidy et al., 2018; Hull et al., 2017; Mandy, 2019). Some participants noted that in lockdown they experienced less sensory overload, in line with another study of autistic mental health in the pandemic (Oomen et al., 2021).

### Pandemic-related threats to mental health

Previous work has shown that variability in how the pandemic is experienced is associated with variability in mental health outcomes during the pandemic (Adams et al., 2021), and our findings support and elaborate this idea. Our qualitative analyses suggest that factors including a lack of connection to others, loss of social support, changes to household roles and dynamics, and family disputes all contributed to mental health declines. Having reduced time and space to self-regulate during lockdown, as well as disruptions to routines and sensory environments, left participants feeling overwhelmed and suffering subsequent mental health declines. Given the unrest caused by COVID-19, plus the association between autism and difficulties with emotional regulation (Mazefsky et al., 2013) and processing (Dijkhuis et al., 2017), our findings are unsurprising. However, with lockdown measures placing restrictions on many people’s ways of coping, the need for additional service support for the autistic community is evident from these results.

Pre-pandemic, accessibility was a problem for many autistic people (Pellicano & Stears, 2020), and this issue appears to have been amplified during the pandemic. Our qualitative data indicate that autistic people struggled to get some basic needs met during the lockdown (e.g. access to specific foods). As observed in the general population (Vindegaard & Benros, 2020), this experience left some autistic adults in our sample experiencing more eating disordered behaviour. Given the elevated prevalence of eating disorders in autistic people (Brede et al., 2020; Westwood et al., 2017, 2018), this finding is concerning. Additional measures for the autistic community, such as including them in priority access times for supermarket entry and delivery slots could be a useful source of support.

Autistic people are also more likely to experience barriers to effectively accessing healthcare (Mason et al., 2019). Our regression analysis indicated that changes in access to support significantly predicted changes in depression over time. Furthermore, our qualitative data suggested that challenges such as waiting times for appointments and diagnostic assessments were perceived to have been exacerbated above the pre-existing lengthy delays (Jones et al., 2014). With our results indicating that mental health needs increased for a subgroup of autistic adults during the pandemic (see also Bal et al., 2021; Oomen et al., 2021; Pellicano et al., 2021), it is essential that additional mental health provisions are in place. Due to the pressures on all areas of healthcare during the pandemic, disruptions were likely unavoidable (Maringe et al., 2020; Søreide et al., 2020; Wastnedge et al., 2021). However, with disparities for autistic people accessing healthcare prior to the pandemic, this gap is likely to widen further unless autism-specific provisions are enacted.

One potential way to support autistic people’s access to healthcare is via telehealth. However, our results indicated variability in participants’ telehealth experiences. While a small proportion of participants found telehealth beneficial, barriers to accessibility were experienced by the majority. Our results identified individual-level autism-specific barriers that compounded the accessibility of telehealth, including challenges associated with sensory sensitivities, body awareness, processing speed, and nonverbal communication: findings in line with previous research into general healthcare accessibility for autistic adults (Nicolaidis et al., 2015). Our findings align with previous recommendations that to increase the accessibility of healthcare for autistic people, changes are required at provider- and system-levels (Nicolaidis et al., 2015). Potential alterations that could support autistic people using telehealth are as follows: providing information and resources on how to navigate telehealth in advance of appointments (allowing individuals time to prepare); providers being more aware of potential autism-related barriers when using telehealth; and providers making adaptations (e.g. allowing additional sessions or covering less material during sessions).

Our results also highlighted the lack of clarity in government guidelines regarding COVID-19 (see also Oomen et al., 2021; Pellicano et al., 2021). This experience left participants feeling distressed and, for some, further isolating themselves. Guidelines were felt to be cognitively demanding to understand, with particular pressure placed on executive functions, which have been associated with barriers to healthcare in autistic adults (Mason et al., 2019). The frequency with which government guidance changed, and the lack of time to prepare for changes, was distressing for many participants. Given the association between predictability and general health and well-being for autistic people (Rodger & Umaibalan, 2011), our findings are unsurprising. Our recommendations align with principles outlined by behavioural and social scientists to improve adherence to government messages during the pandemic (Bonell et al., 2020). This includes ensuring guidance is clear, specific and reviewed regularly, which can support people to anticipate possible barriers in advance of any changes. While these recommendations are made for the general population, based on our results, these may be particularly important for the autistic community.

While a relatively small number of participants had experienced job losses at the time our data were collected (i.e. early stages of the pandemic), our results indicated that financial and employment uncertainty contributed to distress for many (see also Goldfarb et al., 2021). In the context of pre-existing low rates of employment and earnings (Roux et al., 2019), as well as poorer educational outcomes (Shattuck et al., 2012) and employment longevity (Taylor et al., 2015), among autistic adults, the economic crisis caused by COVID-19 may further exacerbate disparities. Although research has begun to investigate this area (Goldfarb et al., 2021), to better understand the economic impact on the autistic community, careful monitoring and additional, longitudinal research is required.

## Limitations

Regarding sampling characteristics, the questionnaire-based format of our study, may have presented a barrier to participation for some autistic adults with certain intellectual or language difficulties. Participants were also recruited from support groups and online communities, and were therefore less likely to be totally isolated pre-pandemic. There was also female dominance in our sample, which is common in online research with autistic adults (e.g. Oomen et al., 2021), but contrary to standard conceptions of the autistic population (Geelhand et al., 2019). Such factors affect generalisability of findings and our ability to investigate sex/gender differences, which should be the focus of future research. The absence of a comparison group means we cannot conclude that autistic adults are more or less vulnerable to the effects of pandemic than the general population. Our prospective data covered only two time points, yet data with more time points would allow for the modelling of nonlinear trends and allow the data-driven identification of subgroups defined by distinctive trajectories of mental health. We also acknowledge that results are based on very early stages of the pandemic.

A strength of the study was the use of longitudinal data of mental health experiences prior to the pandemic, allowing us to investigate changes in mental health over time. It is, however, difficult to determine the impact of the COVID-19 pandemic on mental health beyond the early stages of the pandemic. Further longitudinal research is required, while the pandemic is ongoing and once it abates, to determine any long-term effects.

## Conclusion

This research increases our understanding of how UK-based autistic adults’ mental health has been impacted by the COVID-19 pandemic, adding to the limited evidence-base on this topic. Our study supports the value of using prospective data to evaluate changes in mental health over time and highlights the risk of drawing conclusions based solely on retrospective reports. This work also demonstrates the value of a mixed-methods approach, where qualitative analysis can deepen understanding of a phenomenon observed in quantitative data.

Our findings suggest that there was variability in how autistic people responded to the early stages of the pandemic in terms of their mental health, and that there is a subgroup that experienced worsening depression, anxiety and stress. A key factor associated with a better outcome appeared to be the opportunity to shape the environment to fit with the individual’s needs, such that they have comfortable levels of social and sensory input, and opportunities to pursue preferred activities and routines. Conversely, key pandemic-related risk factors for declining mental health appear to be disrupted access to basic needs and services, uncertainty and the economic impact of COVID-19.

## Supplemental Material

sj-docx-1-aut-10.1177_13623613211065543 – Supplemental material for The impact of early stages of COVID-19 on the mental health of autistic adults in the United Kingdom: A longitudinal mixed-methods study Click here for additional data file. Supplemental material, sj-docx-1-aut-10.1177_13623613211065543 for The impact of early stages of COVID-19 on the mental health of autistic adults in the United Kingdom: A longitudinal mixed-methods study by Rebecca Bundy, Will Mandy, Laura Crane, Hannah Belcher, Laura Bourne, Janina Brede, Laura Hull, Jana Brinkert and Julia Cook in Autism

## Notes

1. There were no statistically significant differences in mean autism trait scores (AQ-10) between those who self-identified as autistic (*M* = 7.56) and those with a formal diagnosis (*M* = 8.2), *t*(131) = –1.165, *p* = 0.246, 95% CI = [−1.74, 0.45].

2. Including self-identified autistic people in research is an increasingly common practice (Benevides et al., 2020; Lewis, 2017) and allowed access to a more diverse population, given barriers to accessing diagnoses in adulthood (Huang et al., 2020).

## References

[bibr1-13623613211065543] Adams R. E. Zheng S. Taylor J. L. Bishop S. L. (2021). Ten weeks in: COVID-19-related distress in adults with autism spectrum disorder. Autism, 25, 2140–2145. 33845620 10.1177/13623613211005919

[bibr2-13623613211065543] Alfuraydan M. Croxall J. Hurt L. Kerr M. Brophy S. (2020). Use of telehealth for facilitating the diagnostic assessment of Autism Spectrum Disorder (ASD): A scoping review. PLOS ONE, 15(7), Article e0236415. 10.1371/journal.pone.0236415 PMC7377392 32702017

[bibr3-13623613211065543] Bal V. H. Wilkinson E. White L. C. Law J. K. Consortium S. Feliciano P. Chung W. K. (2021). Early pandemic experiences of autistic adults: Predictors of psychological distress. Autism Research, 14, 1209–1219. 33559334 10.1002/aur.2480 PMC8014774

[bibr4-13623613211065543] Baron-Cohen S. Wheelwright S. (2003). The Friendship Questionnaire: An investigation of adults with Asperger syndrome or high-functioning autism, and normal sex differences. Journal of Autism and Developmental Disorders, 33(5), 509–517. 10.1023/A:1025879411971 14594330

[bibr5-13623613211065543] Benevides T. W. Shore S. M. Palmer K. Duncan P. Plank A. Andresen M.-L. Caplan R. Cook B. Gassner D. Hector B. L. Morgan L. Nebeker L. Purkis Y. Rankowski B. Wittig K. Coughlin S. S. (2020). Listening to the autistic voice: Mental health priorities to guide research and practice in autism from a stakeholder-driven project. Autism, 24(4), 822–833. 10.1177/1362361320908410 32429818 PMC7787673

[bibr6-13623613211065543] Bishop-Fitzpatrick L. Rubenstein E. (2019). The physical and mental health of middle aged and older adults on the autism spectrum and the impact of intellectual disability. Research in Autism Spectrum Disorders, 63, 34–41. 10.1016/j.rasd.2019.01.001 31768189 PMC6876625

[bibr7-13623613211065543] Bonell C. Michie S. Reicher S. West R. Bear L. Yardley L. Curtis V. Amlôt R. Rubin G. J. (2020). Harnessing behavioural science in public health campaigns to maintain ‘social distancing’ in response to the COVID-19 pandemic: Key principles. J Epidemiol Community Health, 74(8), 617–619. 10.1136/jech-2020-214290 32385125 PMC7368244

[bibr8-13623613211065543] Boulter C. Freeston M. South M. Rodgers J. (2014). Intolerance of uncertainty as a framework for understanding anxiety in children and adolescents with autism spectrum disorders. Journal of Autism and Developmental Disorders, 44(6), 1391–1402. 10.1007/s10803-013-2001-x 24272526

[bibr9-13623613211065543] Braun V. Clarke V. (2006). Using thematic analysis in psychology. Qualitative Research in Psychology, 3(2), 77–101. 10.1191/1478088706qp063oa

[bibr10-13623613211065543] Braun V. Clarke V. (2013). Successful qualitative research: A practical guide for beginners. SAGE.

[bibr11-13623613211065543] Braun V. Clarke V. (2019). Reflecting on reflexive thematic analysis. Qualitative Research in Sport, Exercise and Health, 11(4), 589–597. 10.1080/2159676X.2019.1628806

[bibr12-13623613211065543] Braun V. Clarke V. (2020). One size fits all? What counts as quality practice in (reflexive) thematic analysis? Qualitative Research in Psychology, 18, 328–352. 10.1080/14780887.2020.1769238

[bibr13-13623613211065543] Brede J. Babb C. Jones C. Elliott M. Zanker C. Tchanturia K. Serpell L. Fox J. Mandy W. (2020). ‘For me, the anorexia is just a symptom, and the cause is the autism’: Investigating restrictive eating disorders in autistic women. Journal of Autism and Developmental Disorders, 50(12), 4280–4296. 10.1007/s10803-020-04479-3 32274604 PMC7677288

[bibr14-13623613211065543] Cashin A. Buckley T. Trollor J. N. Lennox N. (2018). A scoping review of what is known of the physical health of adults with autism spectrum disorder. Journal of Intellectual Disabilities, 22(1), 96–108. 10.1177/1744629516665242 27623754

[bibr15-13623613211065543] Cassidy S. Bradley L. Shaw R. Baron-Cohen S. (2018). Risk markers for suicidality in autistic adults. Molecular Autism, 9(1), 42. 10.1186/s13229-018-0226-4 30083306 PMC6069847

[bibr16-13623613211065543] Dahiya A. V. McDonnell C. DeLucia E. Scarpa A. (2020). A systematic review of remote telehealth assessments for early signs of autism spectrum disorder: Video and mobile applications. Practice Innovations, 5(2), 150–164.

[bibr17-13623613211065543] Dijkhuis R. R. Ziermans T. B. Van Rijn S. Staal W. G. Swaab H. (2017). Self-regulation and quality of life in high-functioning young adults with autism. Autism, 21(7), 896–906. 10.1177/1362361316655525 27407040 PMC5625847

[bibr18-13623613211065543] Fancourt D. Steptoe A. Bu F. (2021). Trajectories of anxiety and depressive symptoms during enforced isolation due to COVID-19 in England: A longitudinal observational study. The Lancet Psychiatry, 8(2), 141–149. 10.1016/S2215-0366(20)30482-X 33308420 PMC7820109

[bibr19-13623613211065543] Foley K.-R. Pollack A. J. Britt H. C. Lennox N. G. Trollor J. N. (2018). General practice encounters for young patients with autism spectrum disorder in Australia. Autism, 22(7), 784–793. 28683578 10.1177/1362361317702560

[bibr20-13623613211065543] Geelhand P. Bernard P. Klein O. van Tiel B. Kissine M. (2019). The role of gender in the perception of autism symptom severity and future behavioral development. Molecular Autism, 10(1), 16. 10.1186/s13229-019-0266-4 30976383 PMC6439965

[bibr21-13623613211065543] Goldfarb Y. Gal E. Golan O. (2021). Implications of employment changes caused by COVID-19 on mental health and work-related psychological need satisfaction of autistic employees: A mixed-methods longitudinal study. Journal of Autism and Developmental Disorders. Advance online publication. 10.1007/s10803-021-04902-3 PMC7908957 33635422

[bibr22-13623613211065543] Huang Y. Arnold S. R. Foley K.-R. Trollor J. N. (2020). Diagnosis of autism in adulthood: A scoping review. Autism, 24(6), 1311–1327. 10.1177/1362361320903128 32106698

[bibr23-13623613211065543] Hull L. Petrides K. V. Allison C. Smith P. Baron-Cohen S. Lai M. -C. Mandy W. (2017). ‘Putting on my best normal’: Social camouflaging in adults with autism spectrum conditions. Journal of Autism and Developmental Disorders, 47(8), 2519–2534. 10.1007/s10803-017-3166-5 28527095 PMC5509825

[bibr24-13623613211065543] Jacobson N. S. Truax P. (1991). Clinical significance: A statistical approach to defining meaningful change in psychotherapy research. Journal of Consulting and Clinical Psychology, 59(1), 12–19. 10.1037//0022-006x.59.1.12 2002127

[bibr25-13623613211065543] Jones L. Goddard L. Hill E. L. Henry L. A. Crane L. (2014). Experiences of receiving a diagnosis of autism spectrum disorder: A survey of adults in the United Kingdom. Journal of Autism and Developmental Disorders, 44(12), 3033–3044. 10.1007/s10803-014-2161-3 24915932

[bibr26-13623613211065543] Keen D. Webster A. Ridley G. (2016). How well are children with autism spectrum disorder doing academically at school? An overview of the literature. Autism, 20(3), 276–294. 10.1177/1362361315580962 25948598

[bibr27-13623613211065543] Kinnear D. Rydzewska E. Dunn K. Hughes-McCormack L. Melville C. Henderson A. Cooper S.-A. (2020). The relative influence of intellectual disabilities and autism on sensory impairments and physical disability: A whole-country cohort of 5.3 million children and adults. Journal of Applied Research in Intellectual Disabilities, 33(5), 1059–1068. 10.1111/jar.12728 32187783 PMC8641374

[bibr28-13623613211065543] Lai M.-C. Kassee C. Besney R. Bonato S. Hull L. Mandy W. Szatmari P. Ameis S. H. (2019). Prevalence of co-occurring mental health diagnoses in the autism population: A systematic review and meta-analysis. The Lancet Psychiatry, 6(10), 819–829. 10.1016/S2215-0366(19)30289-5 31447415

[bibr29-13623613211065543] Lewis L. F. (2017). A mixed methods study of barriers to formal diagnosis of autism spectrum disorder in adults. Journal of Autism and Developmental Disorders, 47(8), 2410–2424. 10.1007/s10803-017-3168-3 28516422

[bibr30-13623613211065543] Liptak G. S. Kennedy J. A. Dosa N. P. (2011). Social participation in a nationally representative sample of older youth and young adults with autism. Journal of Developmental & Behavioral Pediatrics, 32(4), 277–283. 10.1097/DBP.0b013e31820b49fc 21285894

[bibr31-13623613211065543] Lovibond P. F. Lovibond S. H. (1995). The structure of negative emotional states: Comparison of the Depression Anxiety Stress Scales (DASS) with the Beck Depression and Anxiety Inventories. Behaviour Research and Therapy, 33(3), 335–343. 10.1016/0005-7967(94)00075-U 7726811

[bibr32-13623613211065543] Mandy W. (2019). Social camouflaging in autism: Is it time to lose the mask? Autism, 23(8), 1879–1881. 10.1177/1362361319878559 31552745

[bibr33-13623613211065543] Mandy W. Lai M.-C. (2016). Annual research review: The role of the environment in the developmental psychopathology of autism spectrum condition. Journal of Child Psychology and Psychiatry, 57(3), 271–292. 10.1111/jcpp.12501 26782158

[bibr34-13623613211065543] Mandy W. Murin M. Baykaner O. Staunton S. Cobb R. Hellriegel J. Anderson S. Skuse D. (2016). Easing the transition to secondary education for children with autism spectrum disorder: An evaluation of the Systemic Transition in Education Programme for Autism Spectrum Disorder (STEP-ASD). Autism, 20(5), 580–590. 10.1177/1362361315598892 26304678 PMC4887819

[bibr35-13623613211065543] Maringe C. Spicer J. Morris M. Purushotham A. Nolte E. Sullivan R. Rachet B. Aggarwal A. (2020). The impact of the COVID-19 pandemic on cancer deaths due to delays in diagnosis in England, UK: A national, population-based, modelling study. The Lancet Oncology, 21(8), 1023–1034. 10.1016/S1470-2045(20)30388-0 32702310 PMC7417808

[bibr36-13623613211065543] Mason D. Ingham B. Birtles H. Michael C. Scarlett C. James I. A. Brown T. Woodbury-Smith M. Wilson C. Finch T. Parr J. R. (2021). How to improve healthcare for autistic people: A qualitative study of the views of autistic people and clinicians. Autism, 25(3), 774–785. 10.1177/1362361321993709 33910390

[bibr37-13623613211065543] Mason D. Ingham B. Urbanowicz A. Michael C. Birtles H. Woodbury-Smith M. Brown T. James I. Scarlett C. Nicolaidis C. Parr J. R. (2019). A systematic review of what barriers and facilitators prevent and enable physical healthcare services access for autistic adults. Journal of Autism and Developmental Disorders, 49(8), 3387–3400. 10.1007/s10803-019-04049-2 31124030 PMC6647496

[bibr38-13623613211065543] Mazefsky C. A. Herrington J. Siegel M. Scarpa A. Maddox B. B. Scahill L. White S. W. (2013). The role of emotion regulation in autism spectrum disorder. Journal of the American Academy of Child & Adolescent Psychiatry, 52(7), 679–688. 10.1016/j.jaac.2013.05.006 23800481 PMC3719386

[bibr39-13623613211065543] Nicolaidis C. Raymaker D. M. Ashkenazy E. McDonald K. E. Dern S. Baggs A. E. Kapp S. K. Weiner M. Boisclair W. C. (2015). ‘Respect the way I need to communicate with you’: Healthcare experiences of adults on the autism spectrum. Autism, 19(7), 824–831. 10.1177/1362361315576221 25882392 PMC4841263

[bibr40-13623613211065543] Oomen D. Nijhof A. D. Wiersema J. R. (2021). The psychological impact of the COVID-19 pandemic on adults with autism: A survey study across three countries. Molecular Autism, 12(1), 21. 10.1186/s13229-021-00424-y 33658046 PMC7927758

[bibr41-13623613211065543] Park S. H. Song Y. J. C. Demetriou E. A. Pepper K. L. Thomas E. E. Hickie I. B. Guastella A. J. (2020) 020/09/01/).Validation of the 21-item Depression, Anxiety, and Stress Scales (DASS-21) in individuals with autism spectrum disorder. Psychiatry Research, 291, 113300. 10.1016/j.psychres.2020.113300 32763554

[bibr42-13623613211065543] Pellicano E. Brett S. den Houting J. Heyworth M. Magiati I. Steward R. Urbanowicz A. Stears M. (2021). COVID-19, social isolation and the mental health of autistic people and their families: A qualitative study. Autism. Advance online publication. 10.1177/13623613211035936 34362263

[bibr43-13623613211065543] Pellicano E. Stears M. (2020). The hidden inequalities of COVID-19. Autism, 24(6), 1309–1310. 10.1177/1362361320927590 32423232

[bibr44-13623613211065543] Pierce M. Hope H. Ford T. Hatch S. Hotopf M. John A. Kontopantelis E. Webb R. Wessely S. McManus S. Abel K. M. (2020). Mental health before and during the COVID-19 pandemic: A longitudinal probability sample survey of the UK population. The Lancet Psychiatry, 7(10), 883–892. 10.1016/S2215-0366(20)30308-4 32707037 PMC7373389

[bibr45-13623613211065543] Ralph L. J. Foster D. G. Rocca C. H. (2020). Comparing prospective and retrospective reports of pregnancy intention in a longitudinal cohort of U.S. women. Perspectives on Sexual and Reproductive Health, 52(1), 39–48. 10.1363/psrh.12134 32189427 PMC8126343

[bibr46-13623613211065543] Rodger S. Umaibalan V. (2011). The routines and rituals of families of typically developing children compared with families of children with autism spectrum disorder: An exploratory study. British Journal of Occupational Therapy, 74(1), 20–26. 10.4276/030802211x12947686093567

[bibr47-13623613211065543] Roux A. M. Garfield T. Shattuck P. T. (2019). Employment policy and autism: Analysis of state Workforce Innovation and Opportunity Act (WIOA) implementation plans. Journal of Vocational Rehabilitation, 51, 285–298. 10.3233/JVR-191046

[bibr48-13623613211065543] Søreide K. Hallet J. Matthews J. B. Schnitzbauer A. A. Line P. D. Lai P. B. S. Otero J. Callegaro D. Warner S. G. Baxter N. N. Teh C. S. C. Ng-Kamstra J. Meara J. G. Hagander L. Lorenzon L. (2020). Immediate and long-term impact of the COVID-19 pandemic on delivery of surgical services. British Journal of Surgery, 107(10), 1250–1261. 10.1002/bjs.11670 32350857 PMC7267363

[bibr49-13623613211065543] Salari N. Hosseinian-Far A. Jalali R. Vaisi-Raygani A. Rasoulpoor S. Mohammadi M. Rasoulpoor S. Khaledi-Paveh B. (2020). Prevalence of stress, anxiety, depression among the general population during the COVID-19 pandemic: A systematic review and meta-analysis. Globalization and Health, 16(1), 57. 10.1186/s12992-020-00589-w 32631403 PMC7338126

[bibr50-13623613211065543] Shattuck P. T. Narendorf S. C. Cooper B. Sterzing P. R. Wagner M. Taylor J. L. (2012). Postsecondary education and employment among youth with an autism spectrum disorder. Pediatrics, 129(6), 1042–1049. 10.1542/peds.2011-2864 22585766 PMC3362908

[bibr51-13623613211065543] Shattuck P. T. Orsmond G. I. Wagner M. Cooper B. P. (2011). Participation in social activities among adolescents with an autism spectrum disorder. PLOS ONE, 6(11), Article e27176. 10.1371/journal.pone.0027176 PMC3215697 22110612

[bibr52-13623613211065543] Taylor J. L. Henninger N. A. Mailick M. R. (2015). Longitudinal patterns of employment and postsecondary education for adults with autism and average-range IQ. Autism, 19(7), 785–793. 10.1177/1362361315585643 26019306 PMC4581899

[bibr53-13623613211065543] Tenzek K. (2017). Negative case analysis. In Allen M. (Ed.), The SAGE encyclopedia of communication research methods (Vol. 3, pp. 1085–1087). SAGE Publications, Inc. 10.4135/9781483381411

[bibr54-13623613211065543] Tomlinson S. R. Gore N. McGill P. (2018). Training individuals to implement applied behavior analytic procedures via telehealth: A systematic review of the literature. Journal of Behavioral Education, 27(2), 172–222.

[bibr55-13623613211065543] Unholz-Bowden E. McComas J. J. McMaster K. L. Girtler S. N. Kolb R. L. Shipchandler A. (2020). Caregiver training via telehealth on behavioral procedures: A systematic review. Journal of Behavioral Education, 29(2), 246–281. 10.1007/s10864-020-09381-7 PMC10479951 37670908

[bibr56-13623613211065543] Valentine A. Z. Hall S. S. Young E. Brown B. J. Groom M. J. Hollis C. Hall C. L. (2021). Implementation of telehealth services to assess, monitor, and treat neurodevelopmental disorders: Systematic review [Review]. Journal of Medical Internet Research, 23(1), Article e22619. https://ovidsp.ovid.com/ovidweb.cgi?T=JS&CSC=Y&NEWS=N&PAGE=fulltext&D=emexb&AN=2010813351 10.2196/22619 PMC7819544 33326409

[bibr57-13623613211065543] Vindegaard N. Benros M. E. (2020). COVID-19 pandemic and mental health consequences: Systematic review of the current evidence. Brain, Behavior, and Immunity, 89, 531–542. 32485289 10.1016/j.bbi.2020.05.048 PMC7260522

[bibr58-13623613211065543] Vogan V. Lake J. K. Tint A. Weiss J. A. Lunsky Y. (2017). Tracking health care service use and the experiences of adults with autism spectrum disorder without intellectual disability: A longitudinal study of service rates, barriers and satisfaction. Disability and Health Journal, 10(2), 264–270. 10.1016/j.dhjo.2016.11.002 27899267

[bibr59-13623613211065543] Vohra R. Madhavan S. Sambamoorthi U. (2016). Emergency department use among adults with autism spectrum disorders (ASD). Journal of Autism and Developmental Disorders, 46(4), 1441–1454. 26762115 10.1007/s10803-015-2692-2 PMC4845033

[bibr60-13623613211065543] Wastnedge E. A. Reynolds R. M. van Boeckel S. R. Stock S. J. Denison F. C. Maybin J. A. Critchley H. O. (2021). Pregnancy and COVID-19. Physiological Reviews, 101(1), 303–318. 32969772 10.1152/physrev.00024.2020 PMC7686875

[bibr61-13623613211065543] Westwood H. Mandy W. Simic M. Tchanturia K. (2018). Assessing ASD in adolescent females with anorexia nervosa using clinical and developmental measures: A preliminary investigation. Journal of Abnormal Child Psychology, 46(1), 183–192. 10.1007/s10802-017-0301-x 28417276 PMC5770498

[bibr62-13623613211065543] Westwood H. Mandy W. Tchanturia K. (2017). Clinical evaluation of autistic symptoms in women with anorexia nervosa. Molecular Autism, 8(1), 12. 10.1186/s13229-017-0128-x 28331571 PMC5356303

[bibr63-13623613211065543] Willig C. (2013). Introducing qualitative research in psychology. McGraw Hill.

[bibr64-13623613211065543] Xiong J. Lipsitz O. Nasri F. Lui L. M. W. Gill H. Phan L. Chen-Li D. Iacobucci M. Ho R. Majeed A. McIntyre R. S. (2020). Impact of COVID-19 pandemic on mental health in the general population: A systematic review. Journal of Affective Disorders, 277, 55–64. 10.1016/j.jad.2020.08.001 32799105 PMC7413844

[bibr65-13623613211065543] Zhou X. Snoswell C. L. Harding L. E. Bambling M. Edirippulige S. Bai X. Smith A. C. (2020). The role of telehealth in reducing the mental health burden from COVID-19. Telemedicine and E-Health, 26(4), 377–379. 32202977 10.1089/tmj.2020.0068

