# Supplementary material for: The impact of early stages of COVID-19 on the mental health of autistic adults in the United Kingdom: A longitudinal mixed-methods study
Source: Autism. 2022 Jan 27;26(7):1765–82. doi: 10.1177/13623613211065543 (PMC9483192; doi:10.1177/13623613211065543)
Supplement: sj-docx-1-aut-10.1177_13623613211065543 – Supplemental material for The impact of early stages of COVID-19 on the mental health of autistic adults in the United Kingdom: A longitudinal mixed-methods study [file sj-docx-1-aut-10.1177_13623613211065543.docx]

## **Supplementary Material**

*Table S1.*

| *COVID-19 Question* |
| --- |
| 1. In the last 7 days, what social distancing measures were in place in your community (please select all that apply to you). (Multiple choice)   - 1. Events were suspended   2. All schools were closed   3. Non-essential shops were closed   4. Restrictions applied to non-essential movement, and travel (e.g. leaving the house was only permitted to get essential supplies, care for others, work or exercise)   5. Restrictions applied to outside activities (e.g. exercising)   6. Land borders were closed   7. People arriving in the country were required to self-quarantine   8. Other  1. Please let us know in your own words, how easy or difficult it was for you to understand government information on Covid-19. (Open-ended) 2. Please let us know if your own words how easy or difficult it was for you to access information on Covid-19. (Open-ended) 3. Please let us know in your own words how easy it was for you to access / receive financial related Covid-19 supports provided by your government (e.g. income support, mortgage relief). (Open-ended) 4. How well have your usual support services (e.g., mental health services, autism support groups, social care or social services) met your needs during Covid-19? (Rating scale) 5. Please tell us in your own words to what degree your needs have been supported by your usual support services (open-ended) 6. To what extent has your anxiety changed because of the Covid-19 virus? Multiple choice 7. To what extent has your anxiety changed because of the lockdown* measures? Multiple choice 8. To what extent has your stress changed because of the Covid-19 virus? Multiple choice 9. To what extent has your stress changed because of the lockdown* measures? Multiple choice 10. To what extent have your feelings of sadness changed because of the Covid-19 virus? Multiple choice 11. To what extent have your feelings of sadness changed because of the lockdown* measures? Multiple choice 12. Have any of the following factors impacted negatively on your mental health and wellbeing during Covid-19? (Please tick all that apply) (multiple choice – multiple selections allowed)     1. Uncertainty about lockdown measures     2. Current financial pressure     3. Uncertainty about employment and financial circumstances     4. Poor physical health     5. Being ill with Covid-19     6. Someone close to me being ill with Covid-19     7. Fear about getting ill with Covid-19     8. Fear about someone close to me getting ill with Covid-19     9. Increases in caring responsibilities     10. Changes to normal routine     11. Changes to social support     12. Feeling isolated or lonely     13. Changes in the home (i.e. increased time at home with other members of the household) 13. Please tell us in your own words how Covid-19 has impacted your mental health (open-ended) 14. Have you experienced any of the following as a result of Covid-19 (Multiple choice, multiple selections allowed)     1. Lost your job or regular income     2. A major reduction in your income (i.e. due to being furloughed, income being reduced by your employer, being put on leave by your employer, or not receiving enough work shifts)     3. Unable to pay bills/rent/mortgage     4. Evicted/lost accommodation     5. Increased caring responsibilities (i.e. caring for children, vulnerable family members or friends, or ill family members or friends)     6. Unable to access enough/suitable food     7. Unable to access usual support services (i.e. support group, mental health services, employment services)     8. Unable to access medication     9. I or someone close to me has become unwell with Covid-19 15. Please tell us in your own words how these hardships have impacted you (open-ended) 16. Have you found any of the following helpful in supporting your mental health and wellbeing during the Covid-19 emergency? (Multiple choice, multiple selections allowed)     1. Spending time exercising     2. Spending time engaging in leisure activities alone (i.e. reading, games, gardening, puzzles)     3. Spending time engaging in leisure activities with members of your household (i.e. playing games, conversing, cooking)     4. Spending time socialising with other people via phone calls, virtual meetings, text messages, emails etc.)     5. Spending time on self-care for mental health (i.e., engaging in mindfulness, meditation, yoga, journaling)     6. Creating a daily or weekly routine or sticking to an existing daily or weekly routine     7. other 17. Please explain in your own words how these or other factors have helped your mental health and wellbeing during Covid-19 (open-ended) 18. Has your life improved in any way as a result of Covid-19 or lockdown* measures? (open-ended) |

**Table 2.**

**Frequency of COVID-19 Variables included in the regression models**

| **Frequency of variables of regression model** | **Yes** | **No** | **Missing** |
| --- | --- | --- | --- |
| Changes in access to support | 57 (81.4%) | 13 (18.6%) | 17 (24.3%) |
| Uncertainty about lockdown measures | 60 (85.7%) | 10 (14.3%) | - |
| Regular exercising routine | 35 (50%) | 31 (44.3%) | 4 (5.7%) |
| Leisure activities alone | 53 (75.7%) | 13 (18.6%) | 4 (5.7%) |
| Engaging in social activities | 37 (52.9%) | 33 (47.1%) | - |
| Disruption in routine | 36 (51.4%) | 30 (42.9%) | - |
| Established a new routine | 55 (78.6%) | 14 (20%) | 1 (1.4%) |
| Self-care (e.g. yoga/meditation) | 25 (35.7%) | 41 (58.6%) | 4 (5.7%) |

***Note*. Frequency and percentages in brakets for the variables included in the regression model.**

**Table 3.**

**Partial correlations between retrospective and prospective self-reported depression, anxiety and stress controlling for basline DASS-21 subscale**

| Retrospective reports | **Change in DASS-Depression (controlling for pre-pandemic DASS-Depression scores)** | **Change in DASS-Anxiety (controlling for pre-pandemic DASS-Anxiety scores)** | **Change in DASS-Stress (controlling for pre-pandemic DASS-Stress scores)** |
| --- | --- | --- | --- |
| Lockdown sadness | .53*** |  |  |
| COVID-19 sadness | .23*** |  |  |
| Lockdown anxiety |  | .20* |  |
| COVID-19 anxiety |  | .23 |  |
| Lockdown stress |  |  | .45*** |
| COVID-19 stress |  |  | .28*** |

Note. * p < .05, ** p < .01, *** p < .001; Partial correlations between participants’ retrospective perceptions of changes in anxiety, stress and sadness as a result of the Covid-19 virus and lockdown measures and changes in the given DASS-21 subscales for stress, depression and anxiety controlling for pre-pandemic mental health scores as measures on the DASS-21 at Wave 1.
